# Vitamin D deficiency following Billroth II surgery - How much vitamin D is enough?: a case report

**DOI:** 10.1186/1757-1626-3-12

**Published:** 2010-01-08

**Authors:** Eva Sampl, Doris Wagner, Claudia Friedl, Harald Dobnig, Astrid Fahrleitner-Pammer

**Affiliations:** 1 Department of Internal Medicine, Division of Endocrinology and Nuclear Medicine, Medical University Graz, Auenbruggerplatz 15, Graz, 8036, Austria; 2 Department of Surgery, Division of Transplantation Surgery, Medical University Graz, Auenbruggerplatz 29, Graz, 8036, Austria; 3 Department of Internal Medicine, Division of Endocrinology and Nuclear Medicine, Medical University Graz, Auenbruggerplatz 29, Graz, 8036, Austria

## Abstract

**Background:**

Vitamin D deficiency with all its consequences is a global health problem.

**Case Presentation:**

We reported a 62-year-old Caucasian woman with alcohol-related liver cirrhosis (Child class A) and a medical history of Billroth II surgery. Although she has taken an oral dose of 16 800 IU vitamin D daily for six weeks to normalise her 25-hydroxyvitamin D level the raise was only moderate.

**Conclusion:**

High-dose oral or parenteral vitamin D therapy is necessary to gain sufficient 25-hydroxyvitamin D serum levels in patients following gastric surgery.

## Background

Vitamin D deficiency is defined as 25-hydroxyvitamin D serum levels below 20 ng/ml; levels ≥20 and <30 ng/ml indicate an inadequate vitamin D status. Approximately 1 billion people worldwide have 25-hydroxyvitamin D levels below 30 ng/ml[1]. The major source of vitamin D is sunlight, which promotes subcutaneous production of vitamin D under the influence of UVB light; only 15-20% of vitamin D in the body derives from food. Apart from musculoskeletal malfunction (eg. osteomalacia, osteoporosis, muscle weakness, falls), vitamin D deficiency seems to be associated with several other diseases, e.g. cancer and cardiovascular or immunological disorders[2]. Corresponding trials demonstrated that a daily vitamin D intake of 800-1000 IU is needed to achieve and maintain 25-hydroxyvitamin D levels between 70-80 nmol/l, which are considered to be optimal for fracture prevention[3].

## Case Presentation

A 62-year-old female Caucasian was admitted to our endocrinology outpatient clinic for evaluation of osteoporosis. The patient had a history of partial gastrectomy with Billroth II reconstruction due to multiple gastric ulcers 27 years previously, and liver cirrhosis (Child A) of more than 15 years' standing due to a period of alcoholism as well as local breast cancer in 2003. For treatment of portal hypertension and recurrent ascites, she was being treated with propranolol, furosemide and spironolactone.

A fasting blood sample taken the morning after admission produced the following results: elevated liver transaminases (GGT 242 U/l [<38], AST 49 U/l [<30]), but unimpaired liver function. Blood count and other parameters were within normal limits. Specific analysis showed low 25-hydroxyvitamin D (20 ng/ml [30.0-60.0]) with normal serum PTH, but elevated levels of bone-specific alkaline phosphatase (74.3 μg/l [7.1-21.3]) and β-Crosslaps (0.53 ng/ml [0.09-0.44]), reflecting accelerated bone turnover. Standardized x-ray of the spine showed hyperkyphosis but no fractures. DXA measurement at the spine and the hip revealed osteopenia with a T-score of -1.8 SD and -2.0 SD, and osteoporosis at the distal forearm with a T-score of -2.8 SD.

The patient was advised to take an oral dose of 16 000 IU (40 drops) of vitamin D 3 per week combined with a daily supplement of 1000 mg calcium and 800 IU vitamin D 3.

At 6 weeks' follow-up, she reported that she had taken 40 drops of vitamin D 3 daily, resulting in a cumulative intake of 705 600 IU in that period. Blood analysis then showed 35 ng/ml 25-hydroxyvitamin D, with a surprisingly small increase of 15 ng/ml.

## Discussion

Oral treatment with 1000 IU vitamin D daily over a period of 3-4 months raises 25-hydroxyvitamin D by about 10 ng/ml[4]. Based on this, an increase of 168 ng/ml would be expected with a daily vitamin D intake of 16 800 IU. The increase of just 15 ng/ml was a surprise. The history of Billroth II may, however, have had a negative impact on absorption, although the enteral absorption of the fat-soluble vitamin D takes place in the duodenum and the proximal jejunum, sections of the gut that are not removed as part of a Billroth II resection.

The exact mechanism of the development of vitamin D deficiency in patients following gastric surgery is not yet known. Malabsorption due to accelerated intestinal passage, impaired secretion of pancreatic enzymes and bacterial overgrowth may lead to steatorrhea [5] and so to avoidance of food rich in vitamin D, which could also contribute to vitamin D deficiency[6].

## Conclusion

Vitamin D toxicity has recently become a controversial issue. According to the literature, a vitamin D intake of 10 000 IU daily does not cause intoxication in the sense of 25-hydroxyvitamin D levels above 150 ng/ml associated with hypercalcemia and hyperphosphatemia, even in healthy people[1,7]. Some special groups of patients may need even higher doses of supplementation to achieve satisfactory serum vitamin D levels. This case demonstrates that patients with a history of gastrointestinal surgery may need either high-dose oral vitamin D supplementation or even parenteral administration.

## Abbreviations

GGT: gamma glutamyl-transferase; AST: aspartate transaminase; PTH: parathyroid hormone; DXA: dual-energy x-ray absorptiometry.

## Consent

Written informed consent was obtained from the patient for publication of this case report and accompanying images. A copy of the written consent is available for review by the Editor-in-Chief of this journal.

## Competing interests

The authors declare that they have no competing interests.

## Authors' contributions

ES and CF collected the patient data, performed literature search and were contributors in writing this manuscript. DW supervised the patient within her liver disease and analyzed the patient data. HD and AFP were responsible for the management of the patient and revised the manuscript critically for important intellectual content. All authors read and approved the final manuscript.
